# Multilevel and Urban Health Modeling of Risk Factors for Diabetes Mellitus: A New Insight into Public Health and Preventive Medicine

**DOI:** 10.1155/2014/246049

**Published:** 2014-11-06

**Authors:** Longjian Liu, Ana E. Núñez

**Affiliations:** ^1^Department of Epidemiology and Biostatistics, Drexel University School of Public Health, Philadelphia, PA 19104, USA; ^2^Department of Medicine, Drexel University College of Medicine, Philadelphia, PA 19129, USA

## Abstract

This study aimed to apply multidisciplinary analysis approaches and test two hypotheses that (1) there was a significant increase in the prevalence of diabetes mellitus (DM) from 2002 to 2010 in the city of Philadelphia and that (2) there were significant variations in the prevalence of DM across neighborhoods, and these variations were significantly related to the variations in the neighborhood physical and social environment (PSE). Data from the Southeastern Pennsylvania Household Health Surveys in 2002–2004 (period 1, *n* = 8,567) and in 2008–2010 (period 2, *n* = 8,747) were analyzed using a cross-sectional comparison approach. An index of neighborhood PSE was constructed from 8 specific measures. The results show that age-adjusted prevalence of DM increased from period 1 (10.20%) to period 2 (11.91%) (*P* < 0.001). After adjusting age, sex, and survey years, an estimate of 12.14%, 18.33%, and 11.89% of the odds ratios for DM was related to the differences in the neighborhood PSE disadvantage, the prevalence of overweight/obesity, and those with lower education attendance, respectively. In conclusion, prevalence of DM significantly increased from 2002 to 2010 in the city of Philadelphia. In addition to risk factors for DM at personal level, neighborhood PSE disadvantage may play a critical role in the risk of DM.

## 1. Introduction

The prevalence of DM is increasing rapidly worldwide; the total prevalence is projected to reach 21% of the US adult population by 2050. Diabetes and its complications cause substantial loss in length and quality of life [1–4]. In 2007, diabetes cost the US an excess of $174 billion [1, 2]. Philadelphia, with an approximate 1.5 million residents (2010 census), is the largest city in the state of Pennsylvania and ranks as the 5th largest city in the US after New York, Los Angeles, Chicago, and Houston. Of these 5 cities, Philadelphia has the highest proportion of minority populations. Data from 2010 census indicates that the racial/ethnical makeup of the city was 44.2% Black, 39.0% White, 12.5% Hispanics and Latino, and less than 5% all other populations. In 2009 report, Philadelphia also had the highest prevalence of DM (10.7%), hypertension (34.5%), and heart disease (4.5%) and the second highest prevalence of obesity (29.3%, after Houston, 29.7%) among the 5 largest cities [5]. In the state of Pennsylvania, Philadelphia ranks last out of 67 counties on the basis of health measures of chronic disease morbidity, risk factors, and mortality rates according to the National City Rank reports in 2012 and 2013 [6]. DM, as one of the leading causes of morbidity and mortality, has posed a serious public health problem in the city of Philadelphia. However, studies on the burden of DM and its associated risk factors, with specific attention to physical and social environmental disadvantages that may drive the health disparities of DM, are scarce. It is well known that there were huge changes in socioeconomic situation including a great economic recession between 2007 and 2009 in the nation. It is also well known that the prevalence of overweight and obesity, a significant risk factor for DM, becomes a serious public health issue in the nation. However, it is not well known whether there was any significant change in the prevalence of DM in the past decade in the city of Philadelphia and whether excessive burdens of DM consistently occurred in certain disadvantage districts and neighborhoods and underserved populations. The present study used data from the Southeastern Pennsylvania Household Health Survey (SEPA-HHS), the largest population survey in the region [7], to test two hypotheses that (1) prevalence of DM significantly increased from 2002 to 2010 in the city of Philadelphia, especially in Blacks, and that (2) there were significant variations in the prevalence of DM across neighborhoods, and these variations were significantly related to the variations in the neighborhood physical and social environment (PSE) disadvantages and the prevalence of people who were overweight/obese. Findings from the study may offer new insights into the control and prevention of DM and risk factors for the city and beyond.

## 2. Research Design and Methods

### 2.1. Design and Population

The SEPA-HHS is conducted biannually by the Public Health Management Corporation (PHMC) using a cross-sectional study design and a probability sample of over 10,000 households from five counties in Southeastern Pennsylvania (Philadelphia, Bucks, Chester, Delaware, and Montgomery counties) [7]. Participants aged 18 years and older are selected randomly using the “last birthday” method, and their health status, health behaviors, and neighborhood environment are obtained via interview using a random-digit dial telephone method. All survey questions are standardized and most have been administered and tested in national health surveys, including items from instruments developed by the National Center for Health Statistics (NCHS) for the Behavioral Risk Factor Surveillance Systems (BRFSS). The survey response (participation) rates vary by years and districts, with an overall response rate between 25% and 30%, which are similar to the national phone survey of BRFSS. The present analysis focused on health disparities across neighborhoods (community level) and DM risk factors (individual/personal level) because Philadelphia has very distinct distributions of residents who live in certain communities or small areas. In the present study, small areas were defined according to zip code classified neighborhoods. Of total 47 five-digit zip codes in Philadelphia, 46 are included in the analysis, as very few residents living in the area with zip code 19112 (*n* < 6) participated in the SEPA-HHS. We used combined deidentified data from SEPA-HHS 2002 and 2004 (total *n* = 8,567 as study period 1) and 2008 and 2010 (total *n* = 8,747 as study period 2) in order to have sample sizes large enough in each of the 46 neighborhoods. In the final sample, the median (interquartile range) sample size of 46 neighborhoods was 180 (121–255) in study period 1 and 211 (118–232) in study period 2.

### 2.2. Study Outcome

In the SEPA-HHS, DM is classified for those who answered* yes* to the question “Have you ever been told by a doctor or other health professional that you have or had diabetes?” Women who have or had DM during pregnancy are excluded in the present analysis.

### 2.3. Measures of PSE at Neighborhood Level

To assess the impact of neighborhood PSE on DM, we created a sum index of PSE from 8 questions of neighborhood measures. These questions addressed (1) access to and usage of recreational facilities; (2) access to fruits and vegetables; (3) quality of accessible groceries; (4) likelihood that neighbors help each other; (5) examples of neighbors working together; (6) sense of belonging; (7) degree of trust in neighbors; and (8) poverty level. Because the strengths of each measure related to DM may be different, we constructed a weighted PSE score through four steps. First, we estimated the relationship (assessed by standard regression coefficients, *SBi*) between 8 PSE measures (*Xi*) and the prevalence of DM (*Y*) using multivariate logistic regression analysis with adjustment for age. Second, the estimated standard *SBi* with a positive relationship to the prevalence of DM was used to weigh the original values of individual measures (*Xi*), resulting in the production of *SBi*∗*Xi*. Third, the weighted measures were summed to create a total PSE score. Lastly, a mean PSE score was calculated for each neighborhood, a higher score indicating a neighborhood with a poorer (disadvantaged) PSE status. The principal of constructing a weighted index has been applied and validated by several studies including our own work [4, 8–11].

### 2.4. Risk Factors at Individual Level

Several demographic, lifestyle, and health status factors were included: age (years), sex (male and female), race/ethnicity (White, Black, and others), and education attendance (<high school, high school, and >high school). Smoking status was identified by affirmative responses to the survey questions “do you now smoke?” or “have you smoked at least 100 cigarettes in your entire life?” Subjects were classified into three groups (current smokers, former smokers, and never smoked). Physical activity status was assessed by responses to the survey questions, “thinking about the past month, how many times per week did you participate in any physical activities for exercise that lasted for at least one half hour, such as walking, basketball, dance, rollerblading, or gardening?” Fruit and/or vegetable consumption were assessed by responses to the survey questions “How many servings of fruits and vegetables do you eat on a typical day?” (a serving of a fruit or vegetable is equal to a medium apple, half a cup of peas, or half a large banana). The prevalence of overweight and obesity was using body mass index (BMI) and calculated using weight (kg) divided by square of height (m). BMI, categorized using the World Health Organization criteria, were underweight (BMI < 18.5 kg/m^2^), normal (BMI: 18.5–24.9 kg/m^2^), overweight (BMI: 25–29.9 kg/m^2^), and obese (BMI ≥ 30 kg/m^2^), respectively.

### 2.5. Statistical Analysis

A serial analysis was conducted on the SEPA-HHS data. First, we described participant characteristics by periods 1 (2002–2004) and 2 (2008–2010). Differences in categorical variables between the two study periods were tested using the Chi-square test and continuous variables using *t*-tests and ANOVA. We mapped age-adjusted prevalence of diabetes across 46 neighborhoods using Geographic Information System (ArcGIS version 10). Age-adjusted prevalence of diabetes was estimated using direct standardization method for the US 2000 population. Second, we used multilevel analysis technique (generalized linear mixed model) to estimate odds ratios of individual-level risk factors (age, sex, race/ethnicity, education level, smoking status, body weight, physical activity, and vegetable and/or fruits intake) and neighborhood-level PSE score for the prevalence of DM [12]. Interaction effects of PSE with periods, PSE with BMI, and PSE with race/ethnicity on the odds of DM were also tested. Finally, 46 neighborhoods were categorized into two groups using the highest quartile of age-adjusted DM prevalence rate (≥13.98%) as the cutoff in order to further estimate the impacts of risk factors on the excess prevalence of DM in neighborhoods with DM rate ≥ 13.98% versus those <13.98%. In the analysis, Model 1 served as the base model and adjusted for age, sex, and survey period. In Models 2 to 6, the same covariates (age, sex, and survey period) were included along with the following additional risk factors: race/ethnicity (Model 2), education attendance (Model 3), behavioral factors (Model 4), overweight/obesity (Model 5), and PSE score (Model 6) in a step-by-step manner. Model 7 tested the association between preventable factors and the odds of DM. Model 8 tested the association between all study factors and DM. Impacts of risk factors on the excess DM rate of neighborhoods with high versus low rates (≥13.98% versus <13.98%) were estimated using the formula (OR_1_ − OR_2_)/(OR_1_ − 1.0) × 100%, where OR_1_ represents OR derived from Model 1; OR_2_ represents OR after adjusting for additional covariate(s); and 1.0 represents OR when there was no excess risk [13].

All statistical analyses were conducted using SAS software version 9.2 (SAS Institute, Cary, NC) [14]. Weighting approach was applied to take into account the probability sampling design of the SEPA-HHS. Multilevel analysis was conducted using SAS Procedure GLIMMIX [12]. A two-sided *P* value ≤ 0.05 was considered as having statistical significance.

## 3. Results

### 3.1. Characteristics of Participants by Periods

Of the 17,254 participants (*n* = 8,507 and *n* = 8,747 in periods 1 and 2), age-adjusted prevalence (95% CI) of DM was 10.20 (9.54%–10.86%) in period 1 and 11.91% (11.27%–12.55%) in period 2. Blacks had the highest DM rates in both periods 1 and 2. The age-adjusted DM rates (95% CI) were 13.96% (12.63%–15.29%) in Blacks, 7.99% (6.97%–9.12%) in Whites, and 8.59% (6.88%–10.29%) in other racial/ethnic groups in period 1 and were 16.09% (14.75%–17.44%) in Blacks, 11.38% (10.21%–12.55%) in Whites, and 10.37% (8.57%–12.18%) in other racial/ethnic groups in period 2.  Table 1 shows significant differences in the means of age and BMI, the proportions of participants by race/ethnicity, and crude prevalence of DM between periods 1 and 2.

### 3.2. PSE Score and Mapping the Prevalence of DM by Neighborhoods and Study Periods

**Figure 1:**
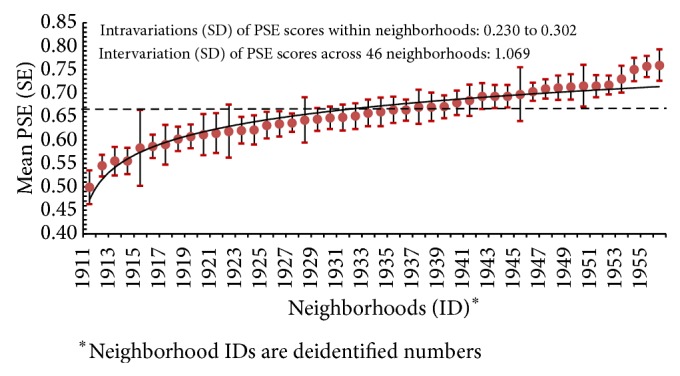
Mean (SE) of physical and social environmental (PSE) scores by neighborhoods.

Logistic regression analysis indicated that among 8 questions of neighborhood PSE measures (*Xi*), 6 were positively associated with the prevalence of DM (*Y*). A sum and weighted PSE score for each participant was created from the production of the 6 corresponding regression coefficients (*SBi*) and their actual measures (*SBi*∗*Xi*). The mean weighted PSE score of 46 neighborhoods was 0.655 (range: 0.499 to 0.760).  Figure 1 shows there were significant variations in mean PSE score across the neighborhoods. The variations (assessed by standard deviation (SD)) of PSE scores within individual neighborhoods ranged from 0.230 to 0.302, which were significantly lower than the variation of PSE score (SD = 1.069) across 46 neighborhoods (*P* < 0.01).

**Figure 2:**
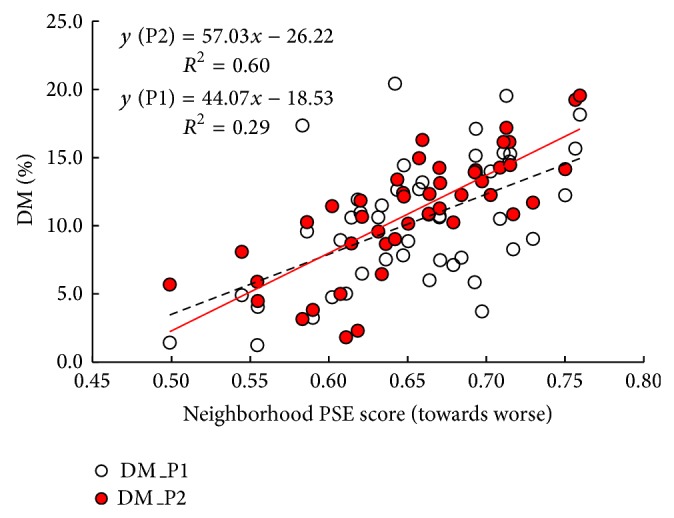
Age-adjusted prevalence (%) of diabetes mellitus (DM) by neighborhoods with different PSE scores for periods 1 and 2.

**Figure 3:**
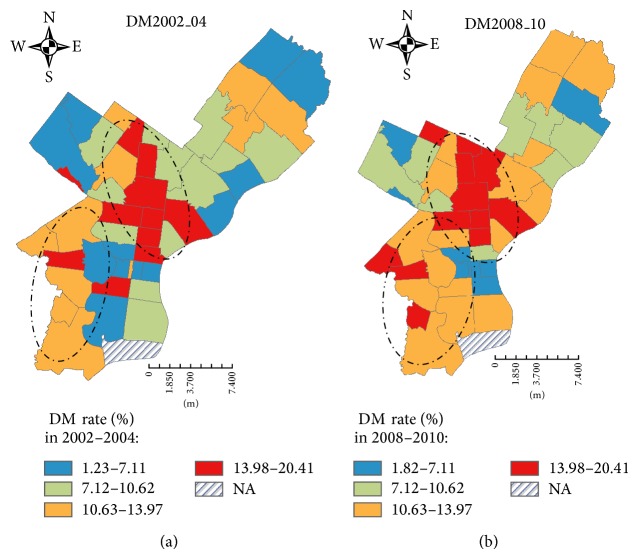
Age-adjusted prevalence (%) of diabetes mellitus by neighborhoods in study periods 1 (a) and 2 (b).

Figure 2 shows that neighborhoods with higher PSE scores (towards a worse PSE status) had higher prevalence of DM in periods 1 (dotted line) and 2 (solid line). Figures 3(a) and 3(b) show significant variations in age-adjusted prevalence of DM across the 46 neighborhoods. In period 1, there were 21 neighborhoods where the prevalence of DM was ≥ 10.6% (i.e., ≥quartile 3 of the disease distribution). These neighborhoods with higher prevalence of DM were predominately located in the North, West, and Southwest districts (Figure 3(a)). In period 2, the number of neighborhoods with diabetes prevalence ≥10.6% increased to 29, a 38.1% increase as compared to period 1 in the city of Philadelphia (Figure 3(b)).

### 3.3. Multilevel Modeling for the Odds of DM

Table 2 shows that aging, being male, being Black, or belonging to other racial/ethnical groups, lower education attendance, and increased BMI were significantly associated with the odds of DM. Subjects in period 2 had 10% higher risk of having DM (OR = 1.10, 95% CI: 1.04–1.17) than those in period 1. Subjects who lived in the neighborhoods with PSE score ≥ 0.70 (quartile 4) had 53% higher risk of having DM than those living in neighborhoods with PSE score <0.62 (Q1). Nonsignificant interaction effects of PSE score on DM were observed.

### 3.4. Excess DM Prevalence Explained by Risk Factors

Model 1 (Table 3) shows that OR (95% CI) of neighborhoods with higher DM rates versus those with lower DM rates was 1.82 (1.63–2.04) (base model). After additionally adjusting for race/ethnicity (Model 2), the OR was reduced to 1.57 (1.40–1.76), a 30.83% reduction from Model 1. When adjusting for education level, an 11.89% reduction was observed in Model 3. When adjusting for overweigh/obesity (Model 5) or PSE (Model 6), a 18.33% or 12.14% reduction of OR was observed, separately. An overall 32.04% reduction of OR was observed when adjusting for all preventable factors (Model 7) and a 51.09% reduction when adjusting for race/ethnicity and preventable factors (Model 8).

## 4. Discussion

The main findings from the study indicate that the prevalence of DM significantly increased from 2002 to 2010 in the city of Philadelphia. Residents who lived in neighborhoods with PSE disadvantages and those with higher prevalence of overweight/obesity and lower education attendance had significantly higher odds of DM. The study is the first to quantitatively examine the association between neighborhood disadvantage and the odds of DM in the metropolitan city Philadelphia of the United States using data from the largest regional household health surveys and using robust data analysis approaches to evaluate health disparities of DM attributable to multilevel and multivariate risk factors.

Most previous studies used census data as a proxy to assess neighborhood PSE, such as using neighborhood poverty rate [8, 15–18]. Using census data may offer an overall estimate of PSE status at community and/or neighborhood level. However, a potential limitation of this approach is that census data may not reflect an individual's actual neighborhood [16]. A better measure of neighborhood PSE should be integrated with the adjustment by individual residents who actually live in the neighborhood [8, 16]. In the present study, we constructed a novel index of neighborhood PSE using multiple neighborhood focused measures and a robust analysis process using standard regression coefficients that is on the basis of a conceptual model of the association between neighborhood PSE and DM [4, 8–11, 19]. This analysis approach adds new evidence to the body of the literature related to DM risk studies. The findings of the study support and reemphasize an important public health and preventive medicine theory that improving neighborhood PSE may play a crucial role in eliminating and reducing health disparities.

An increased prevalence of DM has been observed in most states across the country, including the state of Pennsylvania. The present study adds to the evidence that a 16% increase in age-adjusted DM prevalence was observed from 2002 to 2010 in the city of Philadelphia. Although this unhealthy increased trend was attributable to a number of risk factors, the present study highlights that the prevalence of overweight/obesity, neighborhood disadvantage, and lower education attendance were the strongest, independent, and preventable predictors for the odds of DM. It should be also noted that more than 30% of the variances for neighborhoods with higher DM rates versus lower DM rates could be explained by the variances in the distributions of race/ethnicity (Table 3). Therefore, neighborhood-based and culturally specific-based health promotion programs are requested to control the prevalence of DM at individual and community levels.

The mechanisms by which neighborhood disadvantage produces a higher risk of the prevalence of DM remain to be explored. Potential DM-inducing characteristics of neighborhoods, including limited accessibility to healthcare resources, health food markets, and safe environments, may explain the neighborhood-DM association. Further longitudinal epidemiological studies are requested to test this possible cause-effect association [8, 15].

The present study has several advantages. First, the results provide timely evidence of the increased burden of DM and its association with neighborhood disadvantage. Second, the study contributes to the emerging literature of the application of using weighted sum index to evaluate PSE status and using multilevel analysis techniques to examine disease risk factors at different levels [8, 15, 16, 18–20]. This analysis approach is able to add new evidence to health policy planning, decisions, and preventive medicine. Several limitations should also be kept in mind when interpreting the present findings. First, the results are based on surveys with a cross-sectional study design. Therefore, any casual relationship cannot be interpreted. Second, the classifications of neighborhoods are on the basis of zip codes that may cause misclassification because some small areas within a zip code may have huge differences in PSE. If so, this will lead to an underestimate of the PSE-DM association because the mean value of PSE may not represent this specific zip code's PSE. However, we believe this bias would be very small because each zip code is relatively very distinct by socioeconomic and health status in Philadelphia. Third, DM was classified on the basis of participants' awareness of physician-diagnosed DM. It would lead to an underestimate of DM rate because individuals with unknown DM were not detected. We are unable to test the bias due to lack of serum measures that are needed to diagnose incidence of DM. Last, but not least, the increased trend in the prevalence of DM may partly contribute to the economic recession between the study periods. We are unable to test this potential contribution because the relevant data is unavailable.

In spite of the limitations aforementioned, findings from the present study indicate the accurate situation that the prevalence of DM significantly increased from 2002 to 2010 in the city of Philadelphia. The study, using data from the regionally largest health surveys, suggests that an increased odd of DM is significantly associated with neighborhood disadvantage in a large urban city, as well as individuals with overweight/obesity and lower education attendance.

## Figures and Tables

**Table 1:** Participants characteristics by age, sex, race/ethnicity, and the prevalence of diabetes mellitus for survey years 2002–2004 and 2008–2010.

Variables	2002–2004		2008–2010		
	(*n* = 8,567)		(*n* = 8,747)		*P* value
	Mean, %	(SEM/P)^a^	Mean, %	(SEM/P)^a^	
Continuous var., mean (SEM)					
Age, years	45.84	(0.20)	47.21	(0.19)	<0.0001
Body mass index^b^, Kg/m^2^	27.45	(0.07)	28.04	(0.07)	<0.0001
Categorical var., % (SEP)					
Male	44.73	(0.63)	44.78	(0.67)	0.96
Race/ethnicity					
White	45.76	(0.63)	42.98	(0.65)	0.001
Black	40.37	(0.61)	41.23	(0.65)	
Others	13.88	(0.39)	15.79	(0.53)	
Diabetes mellitus	10.52	(0.38)	13.20	(0.41)	<0.0001

^a^SEM: standard error of mean; SEP: standard error of proportion.

^b^Body mass index (BMI): weight (kg)/height (m) ∗ height (m).

**Table 2:** Multilevel analysis of adjusted odds ratios (95% CI) of risk predictors for the prevalence of diabetes mellitus (DM).

Risk predictors^a^	OR	(95% CI)	*P* value
Individual level			
Age, per 10 years	1.61	(1.55–1.66)	<0.0001
Male versus female	1.20	(1.08–1.34)	0.0011
Race/ethnicity (versus White)			
Black	1.66	(1.46–1.89)	<0.0001
Others	1.63	(1.35–1.97)	<0.0001
Education (versus ≥college)			
High school	1.02	(0.90–1.15)	0.785
<High school	1.31	(1.12–1.53)	0.001
Smoking status (versus never)			
Former smokers	0.88	(0.76–1.01)	0.073
Current smokers	0.98	(0.84–1.13)	0.746
Body weight (versus normal)^b^			
Underweight	0.70	(0.38–1.28)	0.241
Overweight	1.92	(1.65–2.25)	<0.0001
Obesity	4.72	(4.06–5.47)	<0.0001
Physical activity (versus no)			
<1 day/week	1.49	(1.28–1.72)	<0.0001
1–3 days/week	1.16	(0.96–1.39)	0.120
≥4 days/week	1.02	(0.89–1.16)	0.828
Veg./fruit intake (versus ≥5 d/w)			
3-4 days/week	0.98	(0.81–1.19)	0.848
<3 days/week	1.06	(0.87–1.30)	0.548
Study periods^c^			
Period 2 versus period 1	1.10	(1.04–1.17)	0.002
PSE index (versus Q1, <0.62)^d^			
Q2 (0.62–<0.66)	1.42	(1.17–1.74)	0.001
Q3 (0.66–<0.70)	1.38	(1.13–1.69)	0.002
Q4 (0.70–0.76)	1.53	(1.25–1.88)	<0.0001

^a^All predictors were adjusted with each other in multilevel modeling.

^b^Underweight, normal, overweight, and obesity are defined by BMI < 18.5 kg/m^2^, 18.5–24.9 kg/m^2^, 25–29.9 kg/m^2^, and ≥30 kg/m^2^, respectively.

^c^Period 1: 2002–2004; period 2: 2008–2010.

^d^PSE index: physical and social environmental index (towards worse).

Quarter 1: PSE index, 0.50–<0.62.

**Table 3:** Odds ratios (95 CI%) for the likelihood of neighborhoods (NBH) with higher prevalence of diabetes mellitus (DM) versus NBHs with lower prevalence of DM.

		NBHs with DM rate		
Models	Adjusted for	13.98% versus <13.98%		% of excess DM rate accounted
		OR	(95% CI)	
M1^a^	Age, sex, and survey years	1.824	(1.63–2.04)	—
M2	M1 + race/ethnicity	1.570	(1.40–1.76)	30.83
M3	M1 + education	1.726	(1.54–1.93)	11.89
M4^b^	M1 + 3 behavior risk factors	1.790	(1.60–2.00)	4.13
M5	M1 + overweight and obese	1.673	(1.49–1.88)	18.33
M6^c^	M1 + PSE index	1.724	(1.54–1.93)	12.14

M7	M3–M6 (all preventable factors)	1.560	(1.39–1.75)	32.04
M8	All covariates in M2–M6	1.403	(1.24–1.58)	51.09

^a^M1: adjusted for age (years), sex (1 = M, 2 = W), and survey years (period 2 versus 1).

^b^M4: 3 behavioral risk factors: smoking status, physical activity, and vegetable/fruit intake.

^c^PSE index: physical and social environmental index (towards worse).
